# A case series and review of canine idiopathic osteonecrosis of the jaw

**DOI:** 10.3389/fvets.2025.1614645

**Published:** 2025-05-27

**Authors:** Amy Rossi, Jamie G. Anderson

**Affiliations:** ^1^BluePearl Tacoma, Tacoma, WA, United States; ^2^School of Dental Medicine, University of Pennsylvania, Philadelphia, PA, United States

**Keywords:** canine, osteonecrosis, jaws, idiopathic, osteoradionecrosis, medication related osteonecrosis of the jaw

## Abstract

Idiopathic osteonecrosis of the jaw in dogs is a rare disease. Research into human osteonecrosis of the jaw has increased considerably in recent years revealing numerous underlying risk factors and comorbidities. The goal of this case series was to evaluate similar risk factors and comorbidities in dogs. The medical records from 10 cases were retrospectively reviewed for patient signalment, diagnostic results and treatment. Most cases had either cone beam or conventional computed tomography performed which allowed a detailed evaluation of maxillofacial structures. In this cohort, lesions had a predilection for the caudal maxilla and ipsilateral zygomatic arch and dental surgery did not always precede development of lesions. More cases and additional diagnostics will be needed to uncover the etiology of this disease.

## Introduction

Osteonecrosis of the jaw (ONJ) is a debilitating disease in people and animals. The first human cases of ONJ were reported in the late nineteenth century in workers in matchmaking factories exposed to high levels of phosphorous vapors (1–3). These patients developed “phossy jaw” which was characterized by exposed necrotic jawbone and resulted in high mortality rates. Following changes to industrialization laws and the development of antibiotics, cases of “phossy jaw” subsided (1). However, in 2003, cases of ONJ associated with the use of bisphosphonates (BP) emerged in the medical literature, and these cases became known as Bisphosphonate Related Osteonecrosis of the Jaws (BRONJ) (4). Since then, additional medications have been reported to cause ONJ, which prompted a name change to Medication Related Osteonecrosis of the Jaw (MRONJ) (5–8).

Similarly, in the early twentieth century, women painting radium onto watch dials developed necrosis of the jaws after using their lips to shape the brush (9). Since then, therapeutic use of radiation to treat head and neck cancers has been developed. Jaw necrosis, osteoradionecrosis (ORN), remains a serious complication.

Together, MRONJ and ORN comprise 60% of human ONJ (10). Therefore, the majority of the human literature regarding risk factors and comorbidities for ONJ concentrates on MRONJ and ORN. Risk factors for the development of MRONJ or ORN include age, sex, steroid use, and genetic factors (5, 11, 12). Although dental surgeries including extractions and implant placement are recognized as a significant risk factor for MRONJ and ORN, it is estimated that 20–50% of human cases occur without an inciting event (3, 7, 11, 13–17). Historically, it was proposed that trauma associated with oral surgery triggered the development of MRONJ; however, new evidence suggests that infection and necrosis exist prior to surgical intervention (15, 18–23). When necessary, the optimal timing of dental extractions in relation to radiation therapy has not been established. Unsalvageable teeth that are not removed prior to radiation therapy significantly increase the risk of ORN (16).

Additional known causes of human ONJ include trauma, herpes zoster (shingles), deep fungal infection and gangrenous stomatitis, although 4% remain idiopathic (10, 24–26). Gangrenous stomatitis with secondary ONJ is reported in underserved communities, primarily affecting individuals with minimal home care (24, 27). Many pets do not receive home or professional dental care so the role of opportunistic infections in the development of veterinary ONJ needs further investigation. Comorbid conditions seen in human ONJ include diabetes mellitus and anemia (5, 28, 29). Less commonly, hypertension, hyperlipidemia, hypothyroidism and a hypercoagulable state have been associated with ONJ cases (29–35).

While much less is known about ONJ in companion animals, a recent case series detailed 14 cases of canine idiopathic ONJ (36). Peralta et al. reported that 13/14 dogs had ONJ in regions of previous dental extractions and all dogs had some form of dental disease (36). An additional 10 cases of canine idiopathic ONJ are reported here. The goal of this series was to identify common comorbidities between human and canine forms of ONJ, to analyze the role of dental surgery and to add to the existing literature with regards to histopathology and imaging findings.

## Materials and methods

### Criteria for selection

Medical records were included from dogs diagnosed with idiopathic ONJ and examined by a board-certified veterinary dentist or resident in dentistry between 2017 and 2024. Cases were enrolled from one of the following hospitals: BluePearl Tacoma, Tacoma, WA, USA, Veterinary Dentistry and Oral Surgery of New Mexico, Algodones, NM, USA, Flower Mound Veterinary Emergency and Specialty Center, Flower Mound, TX, USA or North Florida Veterinary Dentistry, Jacksonville, FL, USA were reviewed. Cases with visible necrotic bone in the oral cavity without a history of electric burn, radiation therapy, maxillofacial trauma, embedded oral foreign bodies, prolonged corticosteroid usage, or those taking drugs known to cause MRONJ were considered to have idiopathic ONJ.

### Procedures

Comprehensive data was collected from the medical records for each case enrolled. This included: breed, age, sex, weight (kilograms), place of primary residence at time of diagnosis, travel history, duration of signs, time to diagnosis, comorbid conditions and history of corticosteroid use. Records from the referring DVM (rDVM) were scrutinized for the clinical signs and imaging findings prior to any surgical intervention. Areas of necrotic bone based on clinical examination and imaging findings were recorded as maxillary or mandibular. When multiple lesions were observed, lesions were counted separately if there was normal mucosa separating areas of exposed bone. Hematology (CBC) and biochemistry profiles were performed and reviewed as part of a standard diagnostic work up for the oral disease including serial results when available. Additionally, the results of all clinicopathologic tests, including thyroid function, urinalysis, bacterial and fungal culture, bone and adjacent soft tissue histopathology were reviewed. Imaging provided by rDVMs (dental and skull radiography, photographic evidence of oral disease) were reviewed when available. Advanced imaging modalities varied by case and included both conventional and cone beam computed tomography (CBCT). In cases where the imaging was not directly available for review, a radiographic report from a radiologist or a description of findings was available.

## Results

### Patient characteristics

Ten dogs diagnosed with idiopathic ONJ were identified retrospectively for inclusion. Records from the rDVM were available and reviewed in eight cases. In two cases, history and previous treatments were relayed by the owner and the records from the rDVM were no longer available. Records from the board-certified veterinary dentist or resident evaluating the cases were reviewed in all cases. The diagnostics performed for each animal are detailed in Table 1.

**Table 1:** Diagnostics performed in 10 dogs presenting to a veterinary dental specialist and diagnosed with idiopathic osteonecrosis of the jaw (ONJ).

Case #	CBC	Chem	Histopathology	Bacterial culture	Imaging
1	xxx	xx	x	NP	DR, CBCT
2	xx	xx	x	AN/A*	DR, CT
3	xx	xx	x	NP	DR
4	x	x	x	A	DR, CBCT
5	x	x	x	AN/A*	DR, CBCT
6	xx	xx	x	AN/A	DR, CBCT
7	x	x	x	NP	DR, CBCT
8	xx	xx	NP	AN/A	DR
9	x	x	x	AN/A*	DR, CT
10	x	x	NP	NP	DR, CBCT

x = performed once; xx = performed twice; xxx = performed 3 times.

AN/A = anaerobic and Aerobic; A = aerobic only; * = on antibiotics at time of culture; NP = not performed; DR = digital radiography; CT = computed tomography; CBCT = cone beam computed tomography.

The median age was 6.5 years (range 4–12 years). Three dogs were neutered males and 7 were spayed females. The median weight was 26.3 kgs (range 3.5–38.9 kgs). Seven distinct breeds were seen. No dog had a known history of travel outside their state of primary residence. Three dogs had a history of atopy, two had historic recurrent urinary tract infections, one was hypothyroid. One dog had transiently received oral steroids for pruritic skin disease 5 years prior to developing clinical signs of ONJ. Three dogs received steroids for the clinical signs of ONJ. One dog was administered a dose of injectable dexamethasone and two were prescribed oral prednisone. Steroid dosing schedules were not available in any case. Table 2 summarizes the patient characteristics.

**Table 2:** Demographic features, clinical signs, and lesion locations for 10 dogs presenting to a veterinary dental specialist and diagnosed with idiopathic osteonecrosis of the jaw (ONJ).

Case #	Breed	Sex	Age (years)	Wt (kgs)	Clinical signs reported by owner	Comorbidities
1	Cocker Spaniel	MN	12	14	ND	Hypothyroidism
2	Terrier Mix	FS	5	3.5	FS, L, ID/E, WL, OP, EO, B	Atopy
3	Bouvier	FS	4	23.6	H	UTI
4	Bouvier	MN	9	31.8	H, WL	None
5	Labrador	FS	9	29	FS (bi), L, EO, EX	None
6	Pitbull	FS	6	34.4	FS, H, L, I/DE, OP, PT	Atopy, UTI
7	Pug	FS	6	8	H, L, I/DE	None
8	English Bulldog	FS	8	32	FS, H, L, OP, PT, EO, ND, X	Atopy
9	Cocker Spaniel	MN	6	11.5	FS	None
10	Labrador	FS	7	38.9	FS	None

FS = female spayed; MN = male neutered; FS = facial swelling; H = halitosis; L = lethargy; I/DE = inappetence/difficulty eating; OP = oral pain; EO = ipsilateral epiphora; WL = weight loss; PT = ptyalism; ND = nasal discharge; B = ipsilateral blepharospasm; EX = ipsilateral epistaxis; X = ipsilateral exfoliated tooth; bi = bilateral.

### History and physical exam findings

Clinical signs, as reported by the pet owner at the time of initial presentation to their rDVM included: facial swelling (*n* = 6), halitosis (*n* = 5), lethargy (*n* = 5), inappetence or difficulty eating (*n* = 3), oral pain (*n* = 3), ipsilateral epiphora (*n* = 3), weight loss (*n* = 2), ptyalism (*n* = 2), ipsilateral nasal discharge (*n* = 2), ipsilateral blepharospasm (*n* = 1), ipsilateral epistaxis (*n* = 1), ipsilateral exfoliated tooth (*n* = 1) (Table 2). The median duration of clinical signs, prior to clinical suspicion of ONJ by a veterinarian, was 12 weeks (range 2–26).

Physical and anesthetized oral exam findings from the rDVM are summarized in Table 3 and included necrotic bone (*n* = 5), mobile teeth (*n* = 4), gingival recession (*n* = 3), oronasal fistula (*n* = 2), gingivitis (*n* = 2), oral ulcers (*n* = 2), exfoliated tooth (*n* = 1), lymphadenopathy (*n* = 1), and masticatory muscle atrophy (*n* = 1). One dog did not have records available from the rDVM but had previous extractions and was referred for a nonhealing extraction site. No animal was noted to be febrile at any time. Blood pressure measurements were not available for review from any dog.

**Table 3:** Oral exam findings and initial treatments at rDVM facilities in 10 dogs diagnosed with ONJ.

Case #	Physical and Oral examination findings rDVM	Imaging rDVM	Initial Tx(s) and result rDVM
1	M, contralateral ONF	NP	Referral
2	M,G, X	DR	XSS, dehiscence
3	NB, GR	DR	Biopsy of bone and tooth
4	OU, LN	NP	Referral
5	NB, A	SR	XSS, biopsy of bone, dehiscence
6	GR, OU	DR	XSS, dehiscence
7	NB, M, GR, ONF	DR	XSS, dehiscence
8	NB, M, G	NP	XSS, dehiscence
9	Unknown	Unknown	XSS, dehiscence
10	NB	photo	XSS, dehiscence

Except for case 1, which had a contralateral ONF, all oral exam findings are specific to the area of the ONJ. NP = not performed; DR = digital dental radiographs; SR = skull radiographs; NB = necrotic bone; M = mobility of teeth; GR = gingival recession; ONF = oronasal fistula; G = gingivitis; OU = oral ulcers; X = exfoliated tooth; LN = lymphadenopathy; A = masticatory muscle atrophy.

**Figure 1:**
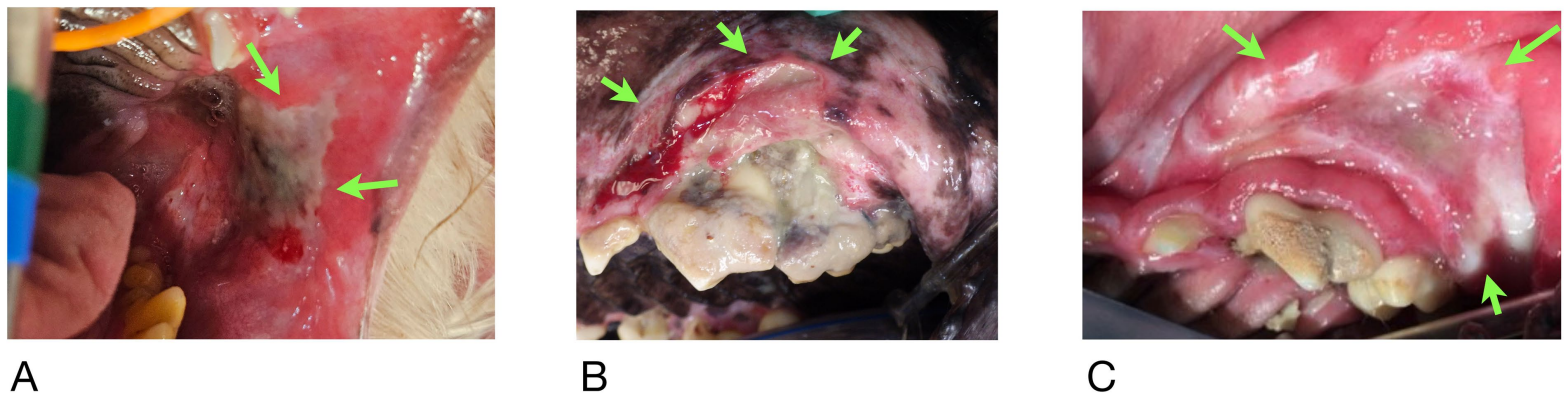
Green arrows demarcating the well-defined irregular ulcerations in Case #2 **(A)**, Case #4 **(B)**, and Case #8 **(C)**.

**Figure 2:**
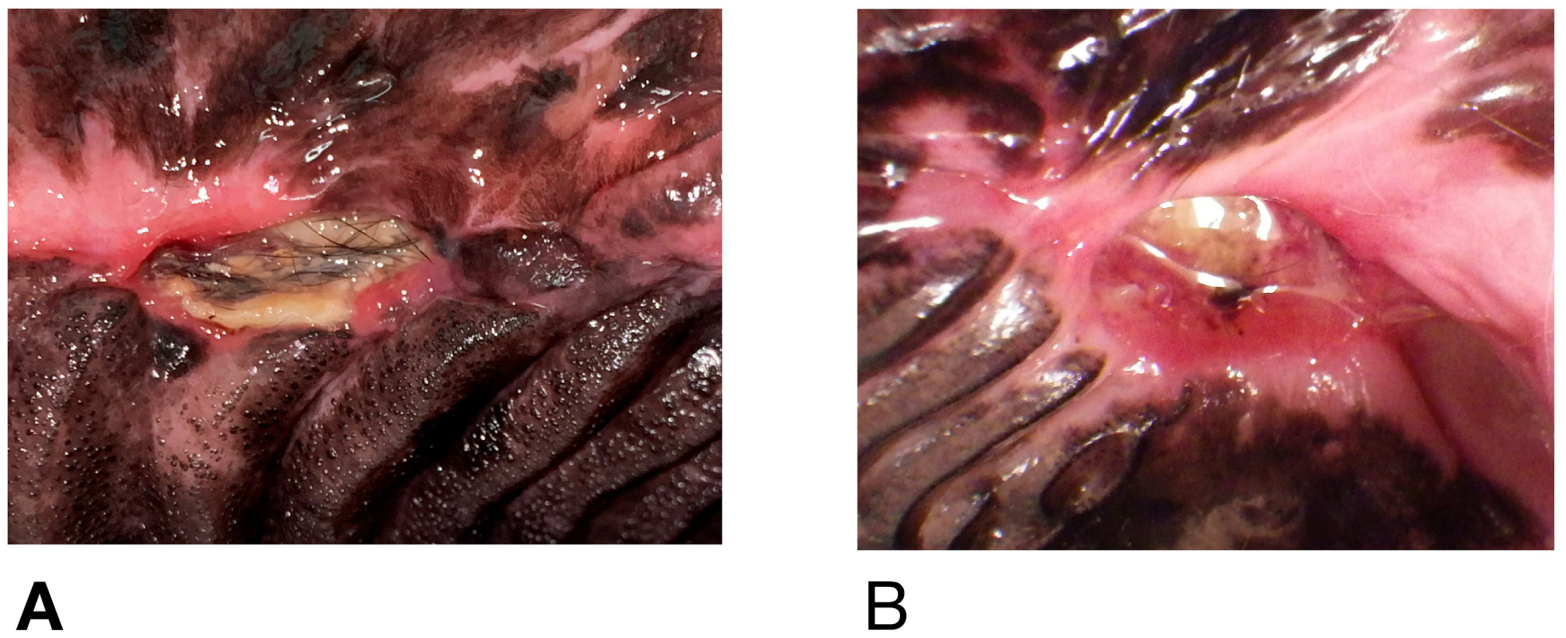
**(A)** Mucosal erythema adjacent to ONJ lesion in Case #5, **(B)** similar lesion in Case #9. Both demonstrate minimal inflammation compared to Figure 1.

At the time of referral to a specialty dentistry clinic, all dogs had static or worsening clinical signs and all dogs had visible necrotic bone on oral exam. Of the dogs that had extractions performed (*n* = 7), all sites had dehisced (Table 3). One dog had decreased retropulsion in the eye ipsilateral to their ONJ lesion. All dogs had erythematous soft tissues surrounding the ONJ regions. Five dogs had erythroleukoplakic ulcerations that were well demarcated with irregular margins (Figure 1). Three of these dogs still had teeth present in the ONJ lesion. Figure 2 demonstrates milder mucosal inflammation associated with ONJ lesions.

### Hematological results

Biochemistry and hematology results were available for review in nine dogs. The tenth dog had specific blood work abnormalities noted in their medical record, but the results were not available for review. Over the course of diagnosis and treatment, hematology was performed 16 times in 10 dogs revealing mild neutrophilia in 4 dogs, monocytosis in 4 dogs, and thrombocytosis in 3 dogs. One dog had a mild nonregenerative anemia. Serum biochemistry was performed 15 times in 10 dogs. The only abnormalities seen more than once were mildly low albumin and mildly elevated globulin levels. A T4 was available in 7 dogs and was below the reference range in two dogs, one of which had confirmatory testing performed. No dogs had diabetes mellitus. A cholesterol level was available in seven dogs and was below the reference range in one. Triglycerides were measured in four dogs and were normal in all four. A lipemia index (3+) was noted in one dog. This data is summarized in the Supplementary Table 1.

### Culture results

Six of ten dogs had bacterial culture and sensitivity performed. Anaerobic and aerobic culture was performed in 5/6 of these dogs, while 1/6 had only an aerobic culture. Three dogs were on antibiotics at the time of culture. *Bacteroides* spp. *and Peptostreptococcus* spp. were the only anaerobic bacteria cultured and both were isolated from the same dog. Aerobic bacteria were cultured from all six animals. Species included *Enterococcus* spp. (*n* = 4), *Escherchia Coli* (*n* = 2), *Staphylococcus Pseudointermedius* (*n* = 2), *Klebsiella Pneumoniae* (*n* = 1), *Proteus Mirabilis* (*n* = 1), *Moraxella* spp. (*n* = 1) *Actinomyces* spp. (*n* = 1), and *Pseudomonas Aeruginosa* (*n* = 1). Five of six dogs had multiple organisms isolated. One dog had only one isolate identified, and it was a methicillin resistant *Staphylococcus Pseudointermedius*. One dog had a fungal culture performed and it was negative.

### Histopathology results

Two dogs did not have biopsies of any tissues. Eight of ten had histopathologically confirmed osteonecrosis. Osteomyelitis (OM) was identified in 5/8 dogs, three of whom were also noted to have bony remodeling. An additional two dogs without OM also had evidence of remodeling. In one dog with OM and one without, the pathologist noted that the pattern seen suggested reactive proliferation prior to necrosis. Six of ten dogs had histopathology performed on soft tissues adjacent to the areas of osteonecrosis. These lesions were characterized by lymphoplasmacytic inflammation in all cases with neutrophils additionally noted in five dogs. One dog had palatal tissue adjacent to the necrotic region that was diagnosed as dysplastic and as chronic active stomatitis (Figure 2B). Three of eight dogs had teeth submitted to the pathologist, but none were examined microscopically.

### Lesion locations

A total of 18 sites of osteonecrosis were clinically identified in ten dogs. Seventeen sites were maxillary (94%) and one dog who initially presented with only a maxillary site later progressed to have a mandibular site. All dogs had at least one maxillary lesion caudal to a canine tooth. Ten of the maxillary sites (56%) were left sided. Twelve of 18 (67%) included a maxillary fourth premolar. The only mandibular site was on the right. At the onset of clinical signs, prior to any dental surgery, thirteen of 18 (72%) had all teeth present in the future ONJ location. At the time of diagnosis, sites of necrosis with no previous extraction and complete dentition were seen in 8/18 (44%) of locations. One location was radiographically missing a tooth with no known history of extractions (Table 4).

**Table 4:** Lesion locations, presence of teeth, imaging findings, treatments applied, and outcome for 10 dogs presenting to a veterinary dental specialist and diagnosed with osteonecrosis of the jaw (ONJ).

Case #	Location	Dentition at onset of signs	Dentition at diagnosis	Sequestrum	Zygoma affected	Treatment	Resolution
1	Rmax PM4	present	present	yes	R, L	S, X	yes
	Rmax PM1-3	Rmax PM2 missing	missing	yes	NA	S, X	yes
2	Lmax PM4-M2	all present	M2 exfoliated, pXSS PM4-M1	yes	L	S, A	yes
3	Lmax PM3 - M2	all present	present	yes	unknown	euthanasia	no
4	Rmax PM3	all present	present	no	no	declined	no
	Lmax PM4-M1	all present	present	no	L	declined	no
	Lmax I1-2	all present	present	no	NA	declined	no
5	Rmax PM4	present	pXSS PM4	no	R	declined	no
	Lmax PM2-4	p XSS PM2, PM3	pXSS PM4	no	L	declined	no
	Lmax I2	missing	missing	no	NA	declined	no
6	Lmax PM4-M1	all present	pXSS M1	no	L	D, X, A	yes
7	Rmax PM1-4	all present	pXSS PM1-PM4	yes	No	S, X, A	yes
8	Rmax PM3 - M2	all present	M1 exfoliated, pXSS PM4, M2	no	R	D, X, A	yes
	Lmax PM4-M1	all present	present	no	unknown	no	yes
	Rman M1	present	present	no	NA	no	yes
9	Lmax M1	p XSS	p XSS	no	L	declined	no
10	Rmax PM4	present	present	yes	No	S	yes
	Lmax PM4	present	present	yes	No	S	yes

S = Sequestrectomy; X = Extractions; D = Debridement of bone; A = Antibiotics; NA = Not applicable; p XSS = Known previous extraction.

### Findings from imaging

**Figure 3:**
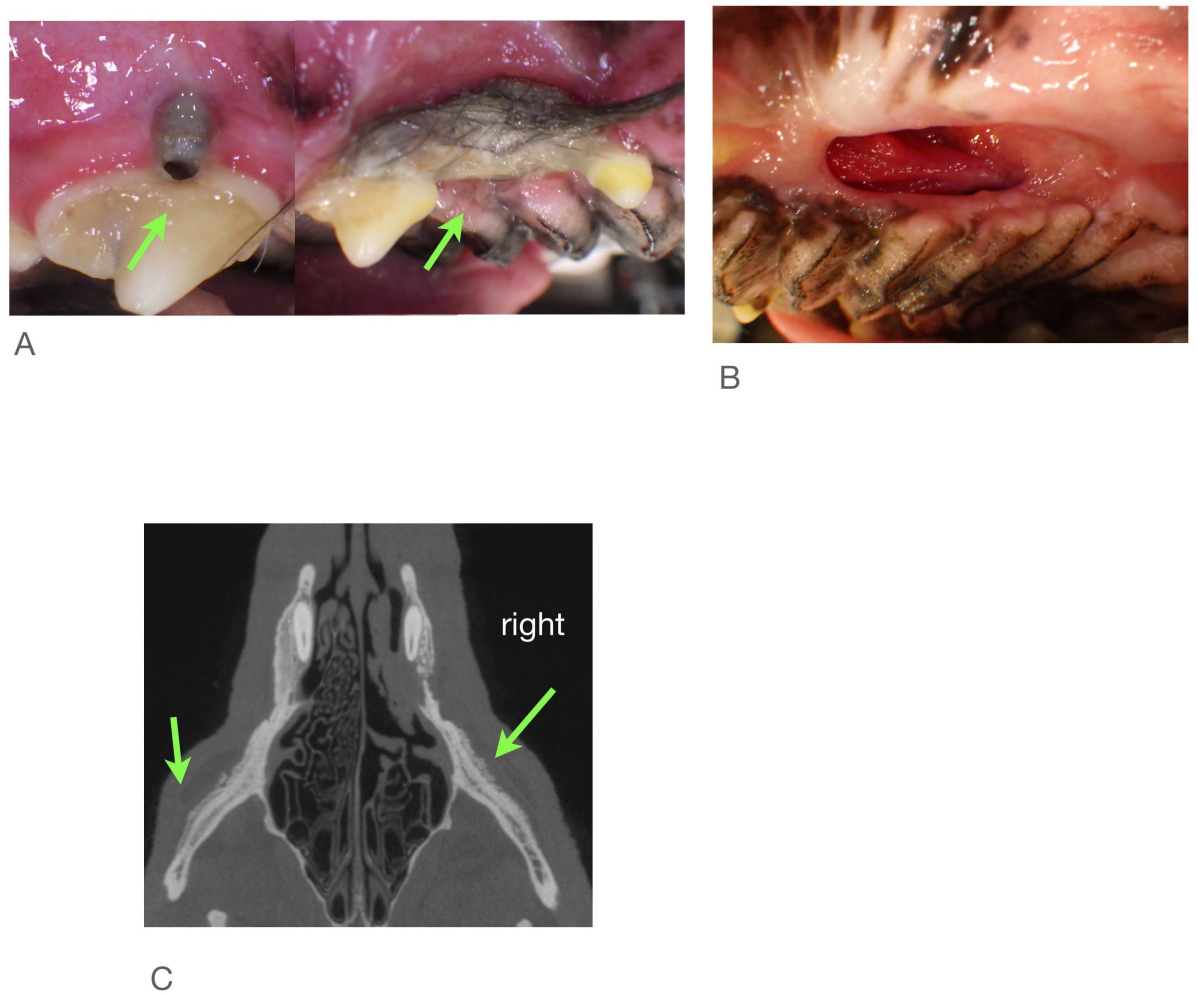
Case #1: stray dog with unknown dental history. Images from first procedure. **(A)** Two locations of ONJ on right. **(B)** Large ONF on left, **(C)** CBCT shows both zygoma affected.

**Figure 4:**
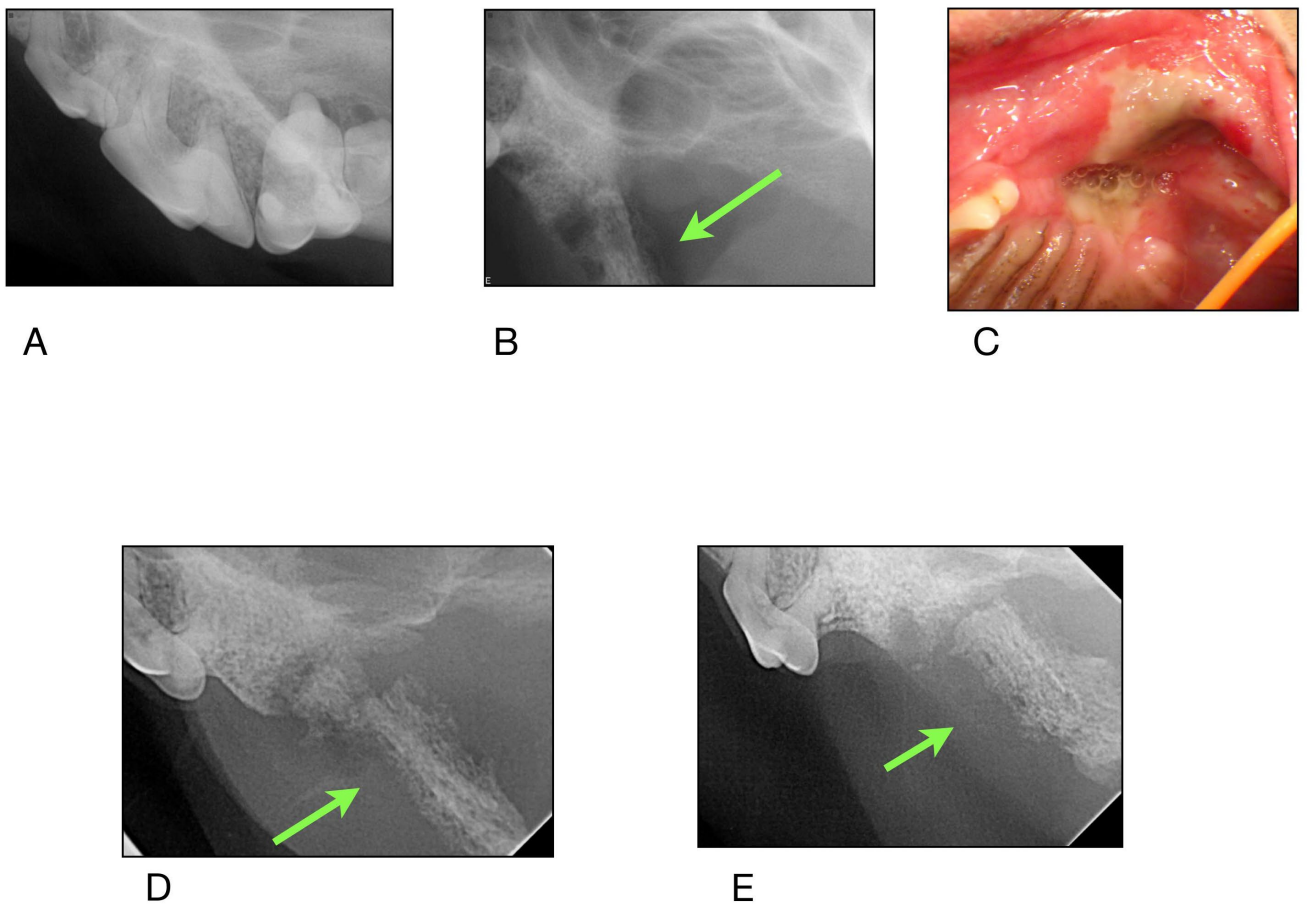
Case #2: **(A)** 11/2023 first procedure pre surgery radiographs: left maxillary second molar exfoliates, left maxillary first molar is extracted, **(B)** 2/2024 second procedure post surgery radiographs: left maxillary fourth premolar has been extracted. Appearance of zygomatic arch (green arrow). **(C)** 5/2024 clinical appearance at time of referral **(D,E)** radiographs of the zygomatic arch at time of referral.

**Figure 5:**
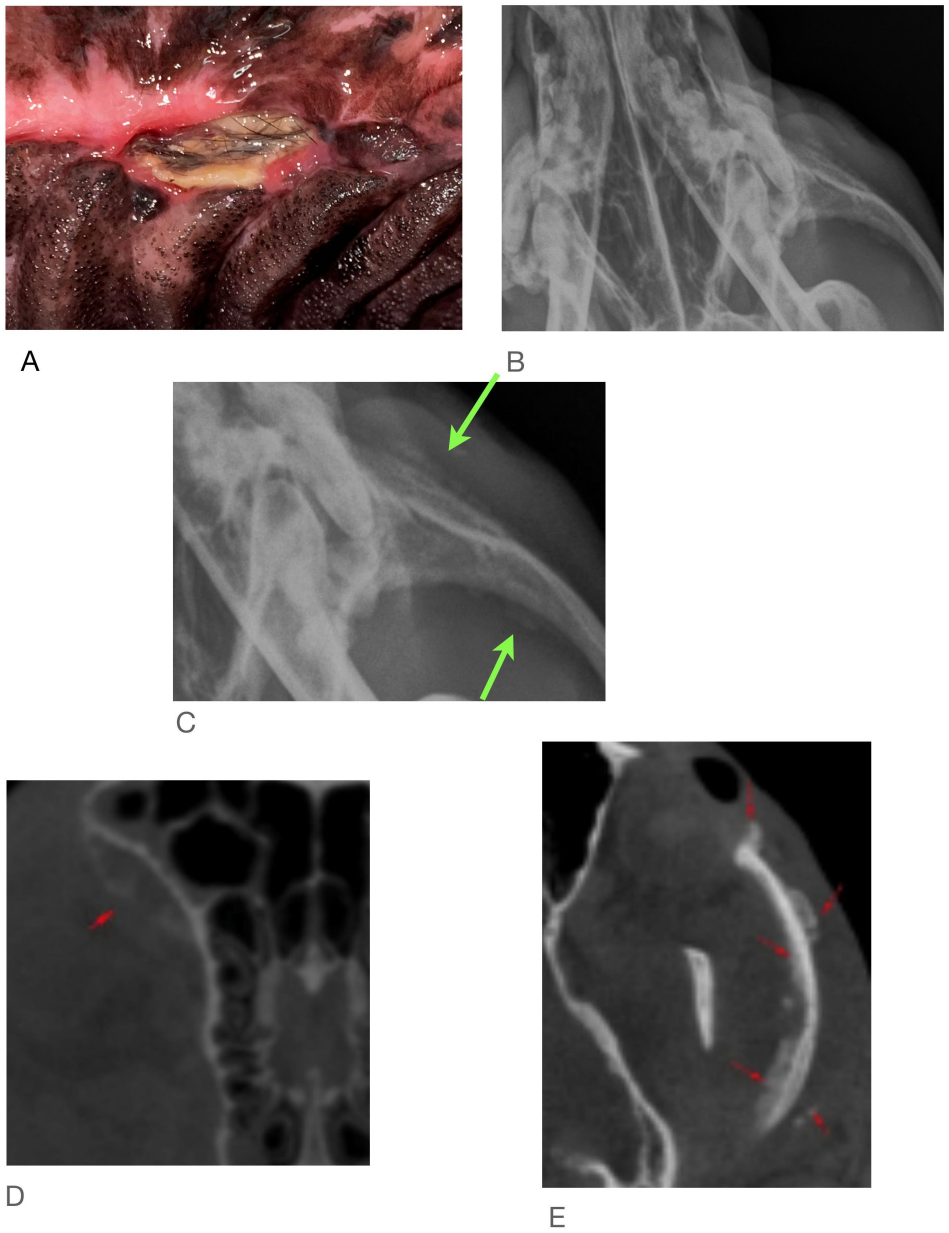
Case #5: **(A)** clinical image, **(B)** skull radiographs demonstrating periosteal reaction on left zygoma, **(C)** close of up area in **A** (arrows), **(D,E)** CT report notes marked heterogeneous bone attentuation of the upper jaw with multiple sites of lysis and new bone formation (red arrows).

**Figure 6:**
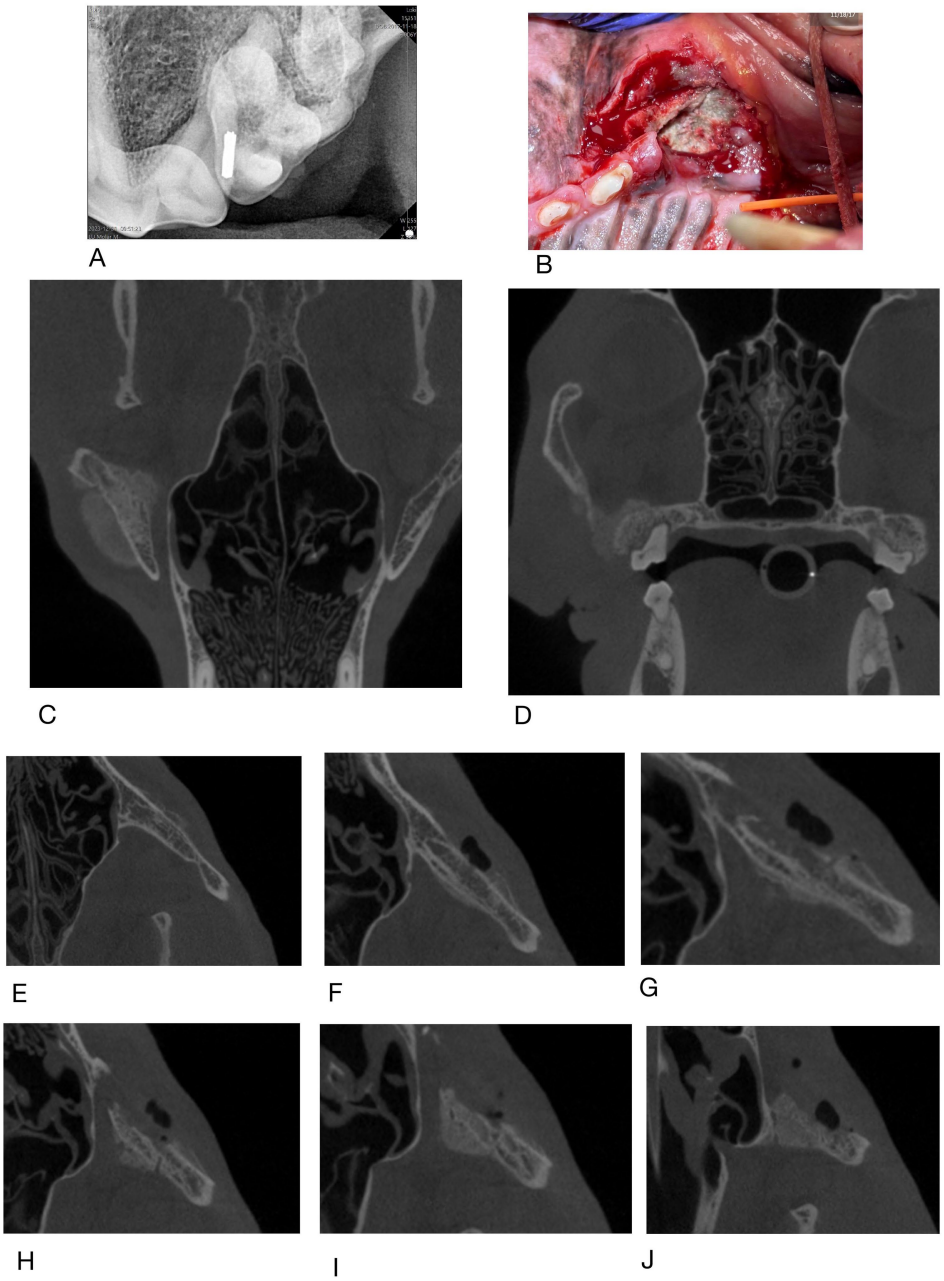
Case #6: **(A)** rDVM radiograph, **(B)** clinical appearance at referral **(C,D)** dorsal and caudal extent of lesion on CBCT in 1/2024, **(E–I)** coronal slices of CBCT taken 2/2024 showing changes to left zygomatic arch moving from dorsal to ventral.

**Figure 7:**
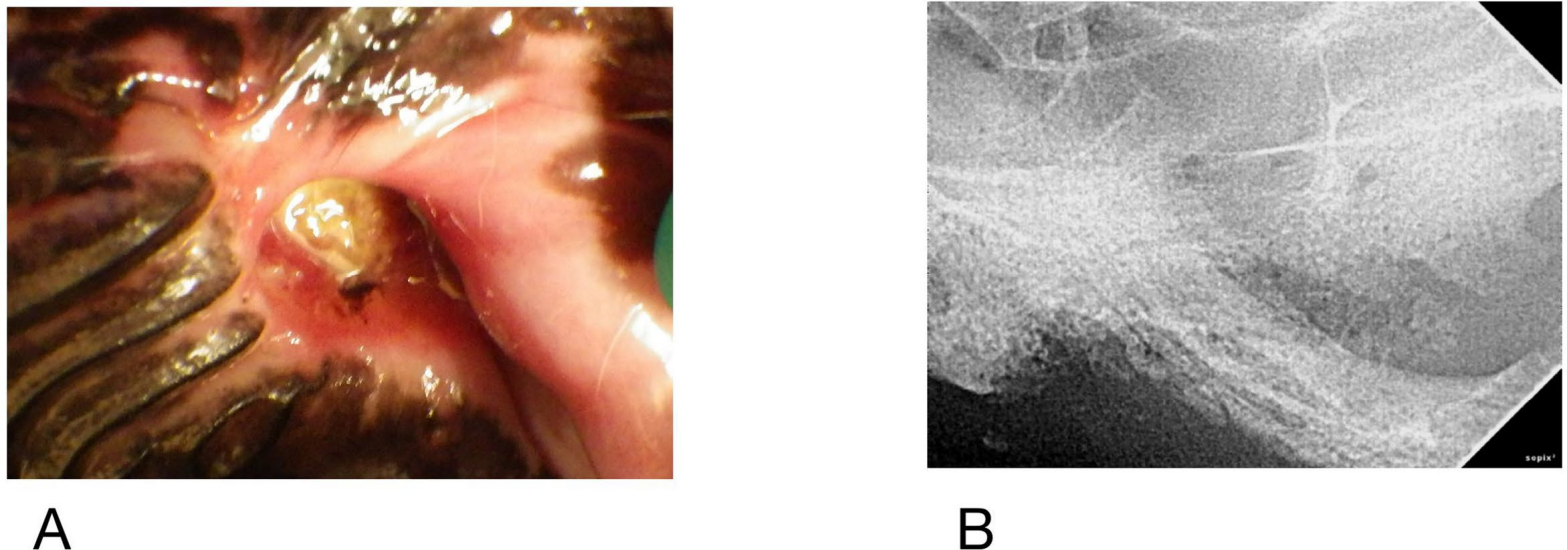
Case #9 **(A)** clinical image of the left maxillary first molar, **(B)** radiograph of region, note periosteal reaction along zygoma.

Examination of all imaging modalities from both rDVM and board-certified veterinary dentists that included the zygomatic arch shows that the ipsilateral zygoma was affected in 7/9 dogs. The radiographic lesions were consistently more extensive than the clinical lesions (Figures 3–7). Interestingly, one dog who had been found recently as a stray had bilateral proliferation of the zygoma and maxilla despite only having a unilateral lesion (Figure 3C). However, this animal was missing most dentition on the left side and had a large left sided oronasal fistula. It is therefore unknown if the teeth exfoliated on their own, if ONJ was previously present, or if extractions had been performed and the area dehisced. A total of 7/18 sites had sequestrum present (Table 4).

### Treatments

Table 4 details the specific treatments and outcomes. The owners of four dogs declined further care beyond biopsy and these animals were euthanized or treated symptomatically until euthanasia was elected.

All dogs received antibiotics for their oral signs. Antibiotics administered included amoxicillin (*n* = 1), cephalexin (*n* = 1), clindamycin (*n* = 8), amoxicillin with clavulanic acid (*n* = 5), metronidazole (*n* = 3), and doxycycline (*n* = 3) and cefovecin (*n* = 1). Three of the dogs that had culture performed were on antibiotics at the time of sampling.

Six dogs had surgery and all experienced resolution of their ONJ. One dog, which had developed ipsilateral ocular signs prior to referral and whose CT showed leakage of vitreous fluid into the oral cavity had enucleation of the left eye. Another dog had an extensive right maxillectomy, including resection of part of the zygoma. Six months later lesions developed in the left caudal maxilla and right mandible of this dog, who was then placed on a regimen of doxycycline, niacinamide and pentoxifylline and was maintained on this regimen but lost to follow up for 4 years. A recheck exam by the same board-certified veterinary dentist 4 years later showed resolution of two sites of necrosis. The right mandibular first molar, which had been a location of necrosis, had been removed at some point, but no further advanced surgeries had been performed.

## Discussion

Given the small sample size of this case series, the data cannot be evaluated statistically and no conclusions about significance can be drawn. In this cohort, no overt association with signalment or comorbid conditions was observed. This differs from what has been previously reported in people where risk factors associated with the development of ONJ include advanced age, female gender, hypertension, anemia, diabetes mellitus, hyperlipidemia, and hypothyroidism (37). Although 7 dogs were spayed females, this sample size is too small to draw any conclusion regarding gender. In the five dogs where triglycerides were measured, they were normal. Blood pressures were not measured independent of anesthesia in any dogs in this report but should be considered in future cases. The most common hematologic abnormalities identified in the present cohort were mild to moderate hypoalbuminemia and hyperglobulinemia, likely secondary to systemic inflammation. One dog was anemic, which might be attributed to anemia of chronic disease as the dog had been diagnosed with hypothyroidism, but further diagnostics were not performed.

Periodontal disease is very common in dogs, but its clinical signs and oral appearance are quite different from this disease (38). Unilateral facial swelling, halitosis, lethargy, oral pain, and difficulty eating were some of the most common signs noted by owners in this case series. Aside from halitosis, most clinical signs of periodontal disease are unrecognized by owners (38). Periodontal disease would also typically be generalized rather than focal. Additionally, in periodontal disease, a layer of connective tissue is always present over resorbed bone (39). Thus, tooth roots are exposed, but either gingiva or mucosa should cover bone. Therefore, facial swelling with a focal, intact, non-discolored mobile tooth should be documented in the medical record. Exfoliation of a single tooth without widespread periodontal disease should also prompt consideration of this disease. Careful evaluation and recording of the state of periodontal tissues should be recorded in the medical record.

Corticosteroid administration has also been previously identified as a risk factor for the development of ONJ, as well as other osteonecrotic conditions such as adult atraumatic necrosis of the femoral and humeral head in people (40). While likely multifactorial in nature, the proposed mechanism for steroids as a predisposing factor relates to reduced blood supply to the bone via steroid-induced hypertrophy and hyperplasia of adipocytes in the marrow (41). The use of high doses of steroids for treatment of COVID-19 has resulted in increased reporting of not just ONJ but also adult avascular atraumatic necrosis of the femoral head (42–44). None of the dogs in this case series were receiving steroids at the time of or immediately prior to the development of clinical signs.

In people with ONJ, results of bacterial culture and sensitivity vary substantially (45, 46). Similarly, in the present cohort of dogs, bacterial culture results were polymicrobial, highly variable, and frequently contained normal oral flora suggesting limited potential pathogenesis of specific organisms. Using molecular detection techniques may be more revealing in future cases. Controversy exists regarding the role of *Actinomyces* in human ONJ (1, 45). Notably, given the debatable role in the human condition, *Actinomyces* were isolated from only one dog in this series.

Both osteonecrosis (8/8 dogs) and osteomyelitis (5/8 dogs) were identified in this cohort of dogs. Histopathology of MRONJ lesions in people do not yield any consistent findings that can distinguish it from either OM or ORN (45). Both necrosis and sequestrum formation can occur with OM, but it is unusual for it to result in exposed oral bone (24). Similarly, this cohort of dogs had no distinguishing histopathology or radiographic findings that could conclusively characterize this disease or distinguish it from osteomyelitis. One prospective study on human bisphosphonate users needing dentoalveolar surgery showed that tartrate-resistant acid phosphatase isoform 5b (TRACP 5b) levels were significantly lower in people who developed necrosis after surgery and it may serve as a useful biomarker of MRONJ development (47). TRACP 5b is interesting because it is an indicator of the number of osteoclasts rather than a measure of osteoclast activity. There are commercially available kits to evaluate TRACP 5b dogs but until these are more widely used, enumeration of osteoclasts per high power field in affected dogs might be informative.

The caudal maxillary location of most lesions in the present study, often including the zygoma, is an interesting finding that conflicts with what is known about the human condition. In people, the mandible is more prone to OM, MRONJ and ORN in part due to its decreased vascularity (5, 48–51). While the reason for difference in ONJ lesion location between species could not be examined in the present study, this might be the result of differences in vascular anatomy between dogs and people, or specifically in dogs that develop ONJ. Interestingly, and more similar to the canine condition, post COVID-19 case reports of ONJ seem to have a maxillary predilection, where one report demonstrated occlusion of a branch of the maxillary artery on CT angiography (30, 42, 43). Although CT angiography is not standardized for the skull in dogs, post contrast evaluation of the vascular supply to the maxilla might be warranted to further explore the underlying mechanism of ONJ in dogs. Even if anatomically normal, another possible vascular cause for ONJ in dogs could be the existence of a hypercoagulable state, with subsequent vascular occlusion and necrosis. In humans and dogs with Legg-Calves-Perthes disease, it is generally accepted that necrosis develops secondary to disruption of the blood supply to the femoral epiphysis (41, 52). Some researchers have proposed that a hypercoagulable/hypofibrinolytic state is responsible for both ONJ and necrotic sites in humans, although this appears contentious (32, 33, 53). Thromboelastography could be considered in dogs with ONJ to better evaluate the coagulation status.

Alternatively, the anatomic prominence of both the zygoma and jugae of the maxillary fourth premolar may predispose these regions to trauma. A subset of human ONJ cases termed “Oral Ulceration Bone Sequestrum” (OUBS) occurs in regions of prominent bone, particularly the mylohyoid ridge. In OUBS, ulceration and sequestrum form after only mild trauma such as laryngoscopy for intubation (54, 55). It is hypothesized that damage to the delicate mucosa results in impaired blood flow and localized necrosis. However, these cases resolve with removal of the sequestrum and most do not last past 8 weeks so in that way are dissimilar to these canine cases (24, 54, 56). There were no reports of any maxillofacial trauma in our patients but given their nonverbal status and periods of unsupervised behavior, this cannot be ruled out. Additionally, trauma combined with suboptimal home care might result in progressive inflammation rather than resolution.

Historically, it was proposed that either trauma associated with the extractions themselves, or the preexisting inflammation/infection were the primary trigger; however, more recent evidence suggests that necrosis in these patients exists prior to surgical intervention (8, 15, 19, 21). The veterinary cases reported here support the idea that ONJ in dogs does not necessarily develop from surgical intervention. Thirteen of eighteen sites had all teeth present at the time of initial clinical signs and 8/18 had all teeth present at the time of diagnosis.

The findings in this case series suggest that dogs with ONJ typically present with facial swelling, halitosis and lethargy. Previous dental surgery may be over emphasized as a risk factor. Further investigation into possible causes include measurement of blood pressure, thromboelastography, examination of extracted teeth to evaluate pulp vitality and periodontal status, enumeration of osteoclasts on histopathology, and angiography. While definitive treatment recommendations have not been established for dogs, the human literature does suggest some possibilities. A10 day pre-surgical course of oral doxycycline and use of a fluorescence lamp during surgery can help determine the location of live versus dead bone (57). Vitamin E and pentoxifylline are currently being investigated in a randomized human prospective trial of MRONJ patients and would be generally considered safe to use in canines (5). Hyperbaric oxygen therapy is another modality that is used in human ONJ that could also be used in dogs (58). When indicated, surgical recommendations include smoothing bone margins, systemic antibiotic use, removing suspect bone and closing the mucosa with sutures (22, 59). Antibiotics are utilized in patients whose signs do not resolve with improved home care and for cases that are poor surgical candidates (5). The use of antibiotics in canine cases is controversial due to polymicrobial culture results which include normal flora as well as the lack of evidence for a primary bacterial cause.

Limitations of this study include the small number of cases and its retrospective nature. In two cases, previous records from the rDVM were not available for direct review. In these cases, the history and previous treatments were obtained solely from the referral record.

## Conclusion

Specific demographic features or comorbid conditions previously associated with ONJ in people were not identified in this cohort of dogs. As such, clinicians should consider this disease as a differential in any animal that presents with systemic or clinical signs beyond what would be expected for periodontal disease alone, such as facial swelling or weight loss, focal exfoliation of a tooth, when dehiscence of oral surgery sites occurs, or when exposed bone is noted in the oral cavity. Advanced imaging frequently involved the zygoma in 7/9 dogs in this case series. Bacterial culture may not be informative. One animal had two sites that appeared to resolve with medical management. Surgical intervention can be successful, resulting in complete resolution of disease even in cases of extensive disease.

## Data Availability

The original contributions presented in the study are included in the article/Supplementary material, further inquiries can be directed to the corresponding author.
